# Characterization of T cell receptors in a novel murine model of nickel-induced intraoral metal contact allergy

**DOI:** 10.1371/journal.pone.0209248

**Published:** 2018-12-17

**Authors:** Yasunari Nakasone, Kenichi Kumagai, Ryota Matsubara, Hiroaki Shigematsu, Kazutaka Kitaura, Satsuki Suzuki, Masashi Satoh, Yoshiki Hamada, Ryuji Suzuki

**Affiliations:** 1 Department of Oral and Maxillofacial Surgery, School of Dental Medicine, Tsurumi University, Yokohama, Japan; 2 Department of Clinical Immunology, Clinical Research Center for Rheumatology and Allergy, Sagamihara National Hospital, National Hospital Organization, Sagamihara, Japan; 3 Center of Oral and Maxillofacial Implantology, Tsurumi University Dental Hospital, Yokohama Japan; 4 Department of Oral and Maxillofacial Surgery, Yokohama General Hospital, Yokohama, Japan; 5 Section of Biological Sciences, Research Center for Odontology, The Nippon Dental University School of Life Dentistry at Tokyo, Tokyo, Japan; 6 Department of Immunology, Kitasato University School of Medicine, Sagamihara, Japan; Hopital universitaire Necker-Enfants malades, FRANCE

## Abstract

Nickel is a component of several alloy types that are widely used in our environment, including several dental alloy types that cause intraoral metal contact allergy. However, metal-specific immune responses in the oral mucosa have not been elucidated because a suitable animal model has not been established. In this study, we established a novel murine model of nickel-induced intraoral metal contact allergy and aimed to elucidate the immune response in terms of T-cell receptor repertoire and cytokine profiles in inflamed oral mucosa. The intraoral metal contact allergy model was induced by two sensitizations of nickel plus lipopolysaccharide solution into the postauricular skin followed by a single nickel challenge of the buccal mucosa. Cytokine expression profiles and T-cell phenotypes were determined by quantitative polymerase chain reaction. T cells accumulated in the cervical lymph nodes and inflamed oral mucosa were characterized by analyzing their T-cell receptor α- and β-chain repertoires, and the nucleotide sequences of complementary determining region 3. Significant swelling and pathological features were histologically evident at 1 day after challenge in mice with nickel allergy. At 1 day after the challenge, CD8-positive T cells producing high levels of T helper 1 type cytokines had accumulated in the allergic oral mucosa. At 7 days after the challenge, excessive nickel allergy in the oral mucosa was suppressed by regulatory T cells. Characterization of the T-cell receptor repertoire in nickel allergic mice revealed the presence of natural killer T cells and T cells bearing *Trav6-6-Traj57* at 1 day after the challenge. Our murine model of nickel-induced intraoral metal contact allergy showed that natural killer T cells and T cells bearing *Trav6-6-Traj57* might be involved in the immune responses of nickel-induced intraoral metal contact allergy.

## Introduction

Nickel (Ni) is a component of several alloy types that are widely used in the environment and it is the most common allergic metal in patch testing [1]. Ni is also a component of several dental alloy types including dentures, orthodontic wires, and dental implants [2]. It was previously suggested that metal allergy in the oral mucosa may be caused by Ni-containing dental alloys [3, 4].

Metal allergy is thought to be an inflammatory disease categorized as a delayed-type hypersensitivity (DTH) reaction caused by haptens that exert antigenicity [5]. Previous studies reported that dental metals may cause allergic reactions in the oral mucosa that manifest as stomatitis, cheilitis, oral lichenoid lesions and burning mouth syndrome [5–8]. However, the pathological mechanism of intraoral metal contact allergy remains unclear because an animal model of metal allergy in the oral mucosa has not been established.

Metal allergy is usually associated with acquired immunity that promotes the trafficking of metal-specific T cells to the site of allergic inflammation [9, 10]. T cells recognize antigens on antigen-presenting cells by T-cell receptor (TCR)s and the high specificity of T cells is determined by the TCRs displayed on their surface, which are heterodimers of an α- and β-chain (TRA and TRB). Previous studies suggested that T cells in the peripheral blood and skin obtained from metal allergy patients had limited TCR repertoires [11, 12].

We previously generated several novel murine models of Ni, palladium (Pd), chromium (Cr), and titanium (Ti)-induced allergic contact dermatitis (ACD) in footpad skin and elucidated their antigen-specific immune responses in terms of TCR usage [13–16]. These models allowed us to identify the accumulation of metal-specific T cells in inflamed skin and clarified that the restricted usage of TCR genes in metal allergy reflects the prolonged exposure of the host immune system to putative metal associated antigens.

The DTH immune response in the oral mucosa differs from that in skin primarily by the difference in local antigen presenting cells and the accumulation of T cells [17, 18]. Previous studies of murine models of DTH in the oral mucosa reported that chemicals such as oxazolone (4-ethoxymethylene-2-phenyloxazol-5-one) and 2,4-dinitro-1-fluorobenzene (DNFB) induced allergic contact mucositis (ACM) in the oral mucosa of mice [17–19]. However, an animal model for metal allergy in the oral mucosa has not been established and therefore the mechanisms of metal-specific immune responses in the oral mucosa have not been elucidated.

Recent advances in next-generation sequencing (NGS) have enabled us to perform quantitative analyses of the TCR repertoire with large amounts of TCR sequencing data. The application of NGS technology to TCRs involved in DTH responses enabled the identification of antigen-specific T cells [20]. In the present study, we generated a novel murine model of Ni-induced allergy in the oral mucosa to explore how accumulated T cells at the site of allergic inflammation contribute to the development of Ni allergy in the oral mucosa and their TCR gene usage.

## Materials and methods

### Ethics statement

All animal experiments in this study were carried out according to the relevant ethical requirements with approval from the committees for animal experiments at Tsurumi University (approval numbers: 27A074 and 30A054) and the Clinical Research Center for Rheumatology and Allergy, Sagamihara National Hospital (approval number: 2010–1). To ensure the health and comfort of all mice, their appearance, behavior, and amount of food and water consumed were monitored daily during experiments. All surgeries were performed under three types of mixed anesthetic agents and all efforts were made to minimize the suffering of animals. To ensure death and prevent pain caused by tissue harvesting, all mice were sacrificed by cervical dislocation under three types of mixed anesthetic agents.

### Animals

*BALB/cAJcl* mice (4-week-old females, n = 66) and *C57BL/6* mice (15-week-old females, n = 8) were purchased from CLEA Japan (Tokyo, Japan). *C57BL/6 CD1d*^*-/-*^ mice (15-week-old females, n = 6) generated by Dr Luc Van Kaer [21], were kindly provided by Dr Masashi Satoh from Kitasato University School of Medicine. During the study period, all mice remained in good health and were allowed to adapt to their environment for one week prior to the commencement of the study. *BALB/cAJcl* mice aged 5 weeks and *C57BL/6* mice aged 16 weeks at the start of an experiment were used. All mice were kept in plastic cages (with a lid made of stainless steel wire) with food and water available *ad libitum*. They were kept in our conventional animal facility with a temperature of 19–23°C, humidity of 30–70% and a 12-h day/night cycle.

### Reagents

NiCl_2_ (>95% pure) was purchased from FUJIFILM Wako Pure Chemical Co., Ltd. (Osaka, Japan). Lipopolysaccharide (LPS) from *Escherichia coli* (O55:B5) prepared by phenol–water extraction was purchased from Sigma-Aldrich (St Louis, MO, USA). NiCl_2_ and LPS were dissolved in sterile saline (Otsuka Normal Saline, Otsuka Pharmaceutical Factory, Inc., Tokushima, Japan).

### Anesthetic agents

The anesthetic was prepared as a mixture of three drugs. Medetomidine hydrochloride was purchased from Nippon Zenyaku Kogyo Co., Ltd. (Fukushima, Japan), midazolam was purchased from Sandoz (Tokyo, Japan) and butorphanol tartrate was purchased from Meiji Seika Pharma Co., Ltd. (Tokyo, Japan). These drugs were kept at room temperature (RT). We mixed medetomidine hydrochloride at a dose of 0.3 mg/kg, midazolam at a dose of 4 mg/kg and butorphanol tartrate at a dose of 5 mg/kg. The concentration ratio of the three types of mixed anesthetic agents was determined according to a previous study [22]. Usually, 0.75 mL of medetomidine hydrochloride, 2 mL of midazolam, 2.50 mL of butorphanol tartrate, and 19.75 mL of sterile saline were mixed to make 25 mL of anesthetic agent. All agents were diluted in sterile saline and stored at 4°C in the dark. The mixed anesthetic agents were administered to mice at a volume of 0.01 mL/g of body weight. All mice were injected intraperitoneally with the mixture of the three types of anesthetic agents.

### Experimental protocol

Based on a previous protocol for the induction of metal allergy in footpad skin [13], we developed a new experimental protocol for the induction of metal allergy in the oral mucosa. Mice were separated into 4 groups: ACM mice, irritant contact mucositis (ICM) mice, control mice, and footpad-ACD (F-ACD) mice with each group consisting of randomly chosen mice. All experiments were carried out in another room after transfer from the animal holding room.

#### Sensitization

A total of 125 μL of 10 mM NiCl_2_ and 10 μg/mL LPS in sterile saline was intradermally injected into mice twice at an interval of 7 days. ACM mice were sensitized at the left and right postauricular skin. The F-ACD mice were sensitized at the left and right regions of the groin. At 7 days after the second sensitization, mice were challenged for the first time.

#### Challenge for elicitation

A total of 25 μL of 10 mM NiCl_2_ without LPS in sterile saline was employed to challenge for elicitation. ACM mice were challenged at the left and right buccal mucosa by submucosal injection. F-ACD mice were challenged at the left and right footpad by intradermal injection. The challenge was repeated twice in ACM and F-ACD mice at an interval of 14 days. Non-sensitized ICM mice were challenged at the left and right buccal mucosa by submucosal injection. Mice sensitized with Ni plus LPS and then challenged with sterile saline were used as a control.

### Measurement of oral mucosa and footpad swelling

Swelling of the buccal area and footpad were measured before challenge and at 6, 12, 24, 48 and 72 h, and 1 week after the first and second challenges using a Peacock Dial Thickness Gauge (Ozaki MFG Co. Ltd., Tokyo, Japan). The thickness of the oral mucosa and footpad were measured before and after challenge and the difference in values was determined. All procedures were performed by the same experimenter with mice under anesthesia.

### Immunohistochemistry

Specimens of buccal mucosa were obtained from control mice, Ni-induced ICM and ACM mice for histological and immunohistochemical (IHC) analyses. Tissue specimens were immersed in 4% paraformaldehyde-lysine-periodate overnight at 4°C. After washing with phosphate buffered saline (PBS) three times for 10 min, fixed tissues were soaked in 5% sucrose/PBS for 1 h at 4°C, 15% sucrose/PBS for 3 h at 4°C, and then 30% sucrose/PBS overnight at 4°C. Tissue samples were snap-frozen in Tissue Mount (Chiba Medical, Saitama, Japan) by immersion into a mixture of acetone and dry ice. Frozen sections were cut into 6-μm thick cryosections and air dried on poly-L-lysine-coated glass slides. For histological analyses, the cryosections were stained with hematoxylin and eosin (HE). For IHC analyses, antigen retrieval was performed and the cryosections were stained with anti-mouse F4/80 (1:1000; Cl-A3-1, Abcam, Cambridge, UK) and anti-CD3 (1:500; SP7, Abcam) monoclonal antibodies (mAbs). F4/80 monoclonal antibody was used to detect mouse macrophage populations in a large number of buccal mucosa tissues. Non-specific binding of mAbs was blocked by incubation of the sections in PBS containing 5% normal goat rabbit serum, 0.025% Triton X-100 (FUJIFILM Wako Pure Chemical), and 5% bovine serum albumin (Sigma-Aldrich) for 30 min at RT. The sections were incubated with primary mAbs for 1 h at RT. After washing three times with PBS for 5 min each, intrinsic peroxidase was quenched using 3% H_2_O_2_ in methanol. After soaking the sections in distilled water, they were washed twice and then incubated with a secondary antibody (biotinylated goat anti-hamster immunoglobulin G or biotinylated rabbit anti-rat immunoglobulin G) for 1 h at RT. After washing three times, the sections were treated with Vectastain ABC Reagent (Vector Laboratories, Burlingame, CA, USA) for 30 min at RT, followed by 3,3-diaminobenzidine staining (0.06% diaminobenzidine and 0.03% H_2_O_2_ in 0.1 M Tris-HCl, pH 7.6; FUJIFILM Wako Pure Chemical). The tissue sections were counterstained with hematoxylin to visualize cell nuclei.

### RNA extraction and cDNA synthesis

Fresh specimens of buccal mucosa were obtained from each mouse and immediately soaked in RNAlater RNA Stabilization Reagent (Qiagen, Hilden, Germany). Total RNA from the buccal mucosa was extracted using the RNeasy Lipid Tissue Mini Kit (Qiagen) according to the manufacturer’s instructions. Complementary DNA (cDNA) was synthesized from DNA-free RNA using the PrimeScript RT reagent Kit (Takara Bio, Tokyo, Japan) according to the manufacturer’s instructions.

### Quantitative polymerase chain reaction

The expression levels of immune response-related genes including T cell-related CD antigens, cytokines, cytotoxic granule, and transcription factors of regulatory T cells were measured by quantitative polymerase chain reaction (qPCR) using the Bio-Rad CFX96 system (Bio-Rad, Hercules, CA, USA). Specific primers for GAPDH, CD4, CD8, IFN-γ, Granzyme B, Foxp3, IL-4, IL-10, NK1.1, and CD1d were described previously [13, 23–25]. Freshly isolated total RNA from specimens of the buccal mucosa and submandibular lymph nodes were converted to cDNA. The PCR consisted of 5 μL SsoFast EvaGreen Supermix (Bio-Rad), 3.5 μL RNase/DNase-free water, 0.5 μL of 5 μM primer mix, and 1 μL cDNA in a final volume of 10 μL. Cycling conditions were as follows: 30 s at 95°C followed by 45 cycles of 1 s at 95°C and 5 s at 60°C. At the end of each program, a melting curve analysis was performed from 65°C to 95°C to confirm homogeneity of the PCR products. All assays were repeated three times and mean values were used to calculate gene expression levels. Five 10-fold serial dilutions of each standard transcript were used to determine the absolute quantification, specification, and amplification efficiency of each primer set. Standard transcripts were generated by the *in vitro* transcription of the corresponding PCR product in a plasmid. The nucleotide sequences were confirmed by DNA sequencing using the CEQ8000 Genetic Analysis System (Beckman Coulter, Fullerton, CA, USA). Their quality and concentration were validated using an Agilent DNA 7500 Kit in an Agilent 2100 Bioanalyzer (Agilent, Santa Clara, CA, USA). GAPDH gene expression was used as an internal control. The expression levels of each target gene were normalized to GAPDH expression.

### Unbiased amplification of TCR genes, amplicon sequencing and assignment of TRV and TRJ segments in TCR genes

Total RNA was prepared from the specimens of the buccal mucosa and cervical lymph nodes from ICM and ACM mice at day 1 after challenge and converted to cDNA with Superscript III reverse transcriptase (Invitrogen, Carlsbad, CA). Next, unbiased adaptor-ligation PCR was used to amplify the TCR genes [26]. High-throughput sequencing was performed with the Illumina Miseq paired-end platform (2 × 300 bp) (Illumina, San Diego, CA, USA). TRV and TRJ segments in TCR genes were assigned a data set of reference sequences from the international ImMunoGeneTics information system (IMGT) database (http://www.imgt.org). Data processing, assignment, and data aggregation were automatically performed using repertoire analysis software originally developed by our group (Repertoire Genesis, Osaka, Japan).

### TCR data analyses

The amino acid sequences of complementarity-determining region 3 (CDR3) regions ranged from a conserved cysteine at position 104 of the IMGT nomenclature to a conserved phenylalanine at position 118 with the following glycine translated from the nucleotide sequences. A unique sequence read was defined as a sequence read having no identity in TRV and TRJ and a deduced amino acid sequence of CDR3 with other sequence reads. The copy number of identical unique sequence reads in each sample was automatically counted by repertoire analysis software and then ranked in order of the copy number. Percentage occurrence frequencies of sequence reads with TRAV, TRAJ, TRBV, and TRBJ genes in total sequence reads were calculated.

### Statistical analysis

Statistically significant differences between the mean values of each experimental group were analyzed using the Kruskal–Wallis test followed by Dunn's multiple comparison tests and Mann–Whitney *U-*test using GraphPad Prism 7 software for Windows (GraphPad Software Inc., San Diego, CA, USA). A *p*-value < 0.05 was considered significant, a *p*-value < 0.01 was considered highly significant, and a *p*-value < 0.001 was considered extremely significant.

## Results

### Ni-induced allergic *BALB/cAJcl* mice develop swelling in the oral mucosa

**Fig 1:**
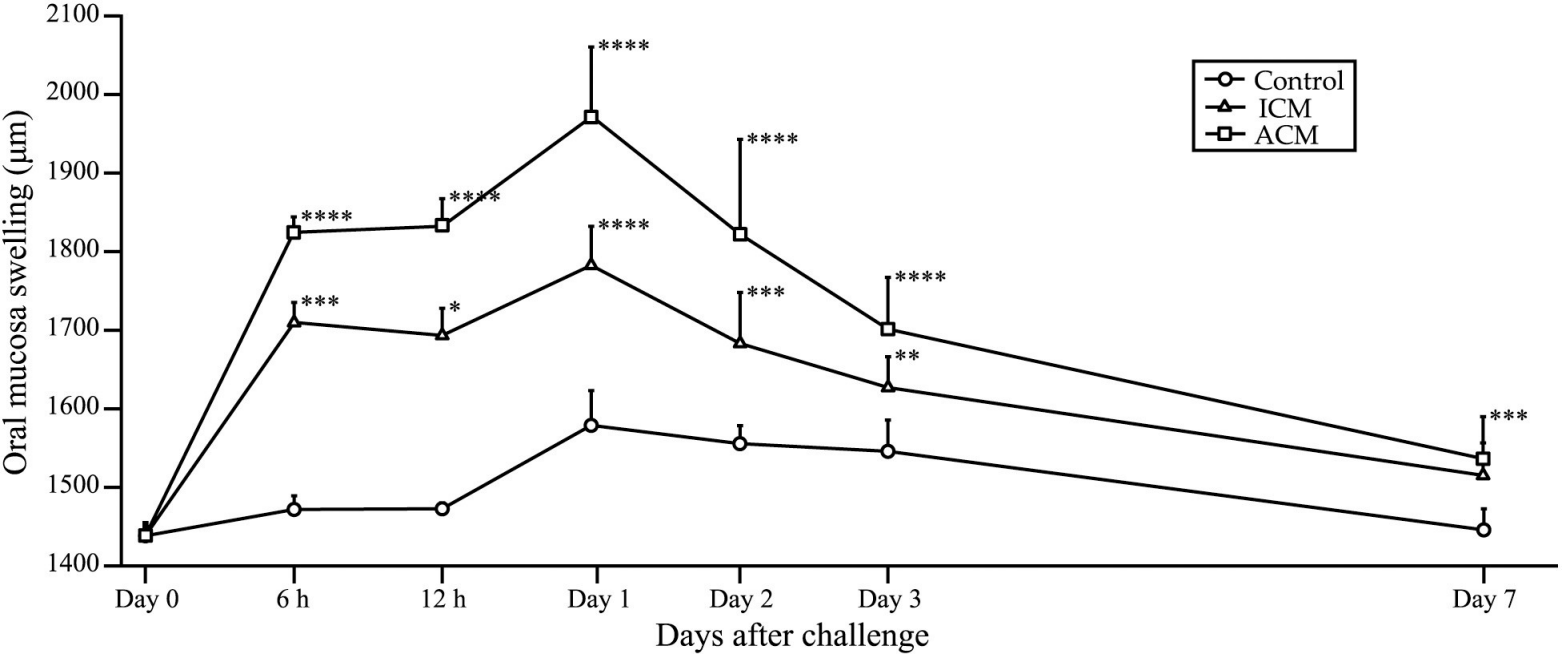
Swelling of the oral mucosa in Ni-induced allergic mice. Buccal area swelling from 6 h to day 7 after challenge was significantly higher in ACM mice compared with control and ICM mice. Bars and error bars indicate mean plus standard deviation. Statistical significance was tested by Dunn's test (* *p* < 0.05, ** *p* < 0.01, *** *p* < 0.001, **** *p* < 0.0001).

**Fig 2:**
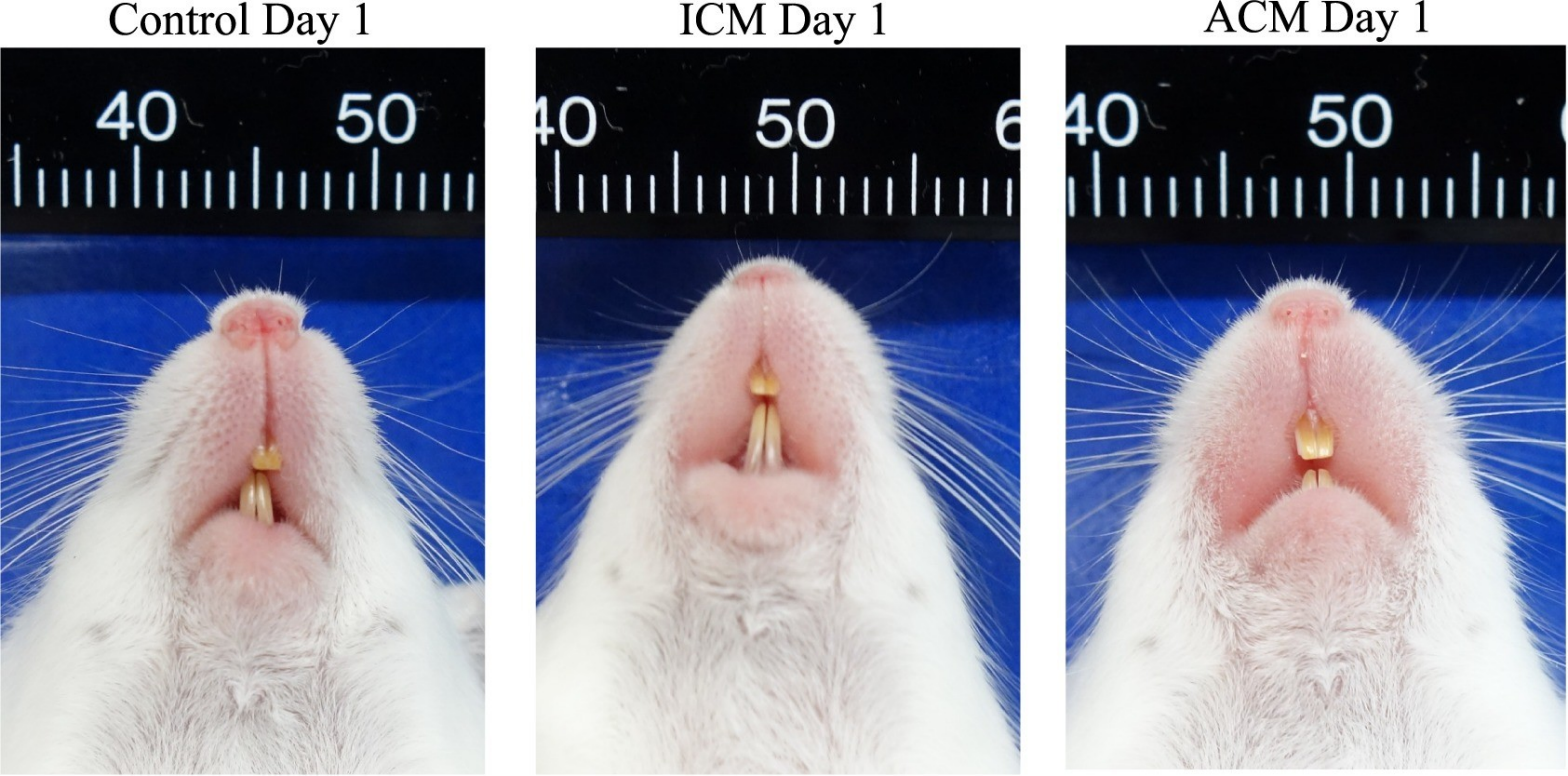
Macroscopic findings of the oral mucosa in Ni-induced allergic mice. Significant redness and swelling are evident in the buccal area of ACM mice compared with control and ICM mice at day 1 after challenge.

Swelling of the buccal area of the oral mucosa of all mice reached a maximum at 1 day after challenge (Figs 1 and 2). At 7 days after challenge, swelling of the buccal mucosa was significantly higher in ACM mice compared with control mice, whereas swelling in ICM mice was not significantly different compared with control mice. From 6 h to 3 days after challenge, swelling of the buccal area in the oral mucosa was increased in ACM mice compared with control and ICM mice (Fig 1).

### Histopathological and immunohistochemical analyses of F4/80 in the oral mucosa of Ni-induced allergic *BALB/cAJcl* mice

**Fig 3:**
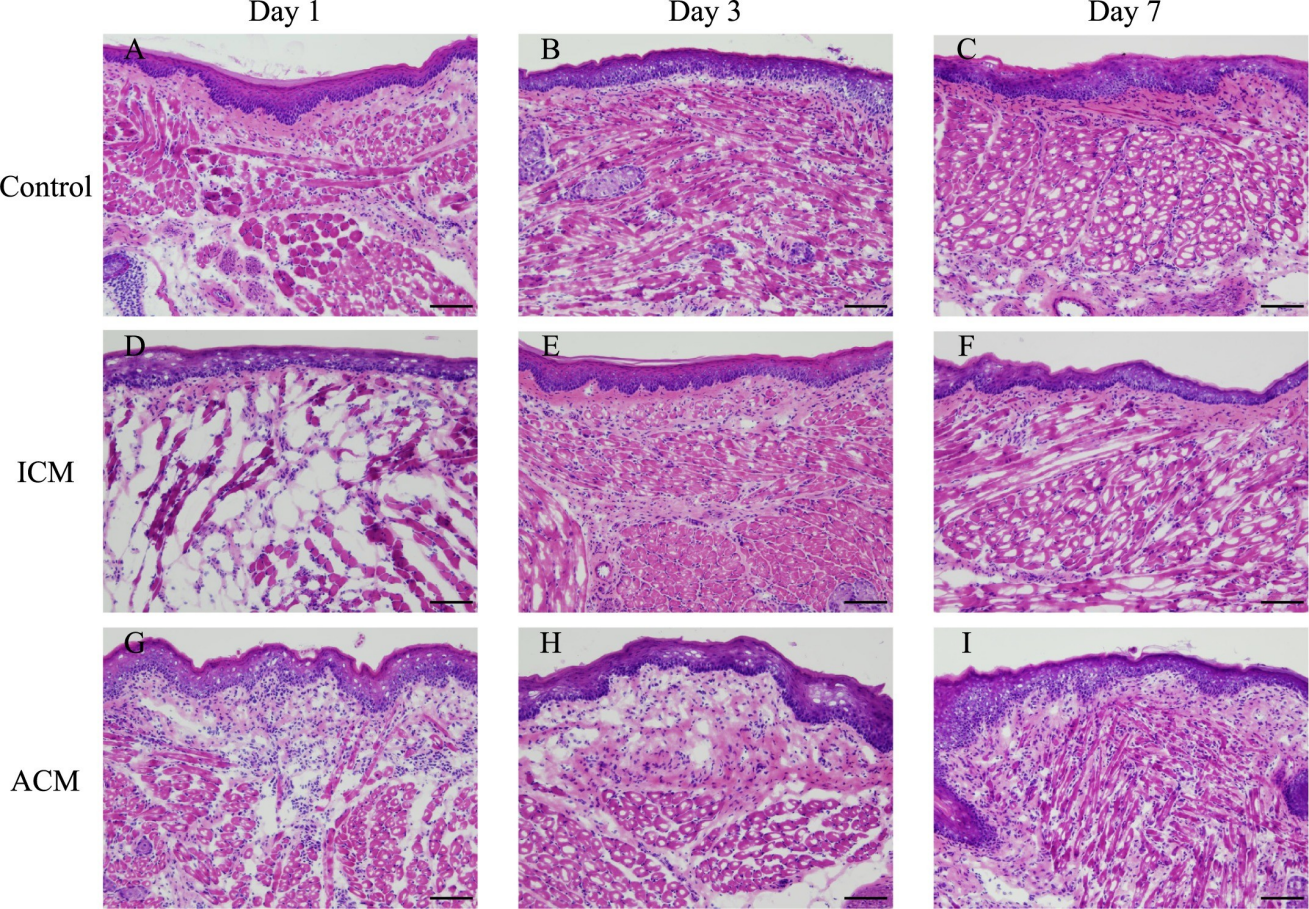
Histopathological analyses of HE staining in the oral mucosa of Ni-induced allergic mice. Oral mucosa tissue sections of each mouse were stained with HE at days 1, 3, and 7 after challenge. At day 1 after challenge, abundant infiltration of mononuclear cells, swelling of the oral mucosal epithelium and epidermal spongiosis were present in ACM mice (G). Scale bar = 10 μm.

**Fig 4:**
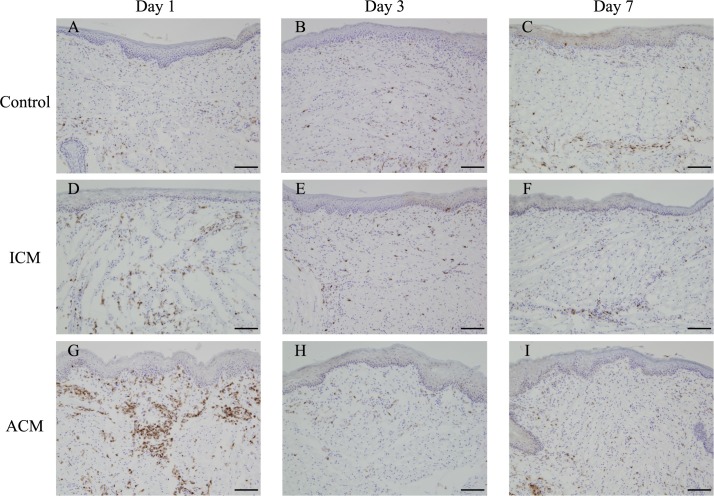
Immunohistochemical analyses of antigen-presenting cells in the oral mucosa of Ni-induced allergic mice. Frozen buccal mucosa sections were stained with anti-F4/80 antibodies at days 1, 3, and 7 after challenge. At day 1 after challenge, abundant infiltration of macrophages into the epithelial basal layer and the upper dermis was observed in ACM mice (G). Scale bar = 10 μm.

We examined the inflammatory changes and infiltration of macrophages into the inflamed oral mucosa of Ni-induced allergy mice. The oral mucosa of Ni-induced ICM and ACM mice, and control mice at 1, 3, and 7 days after challenge were examined. HE staining showed dense inflammatory cell infiltration in the epithelial basal layer and upper dermis, as well as swelling of the oral mucosal epithelium and epidermal spongiosis in ACM mice at 1 day after challenge (Fig 3G). IHC staining showed that F4/80-positive macrophages existed predominantly in the epithelial basal layer and upper dermis of ACM mice at 1 day after challenge (Fig 4G).

### Immunohistochemical analysis of CD3 and expression levels of T cell markers in the oral mucosa of Ni-induced allergic *BALB/cAJcl* mice

**Fig 5:**
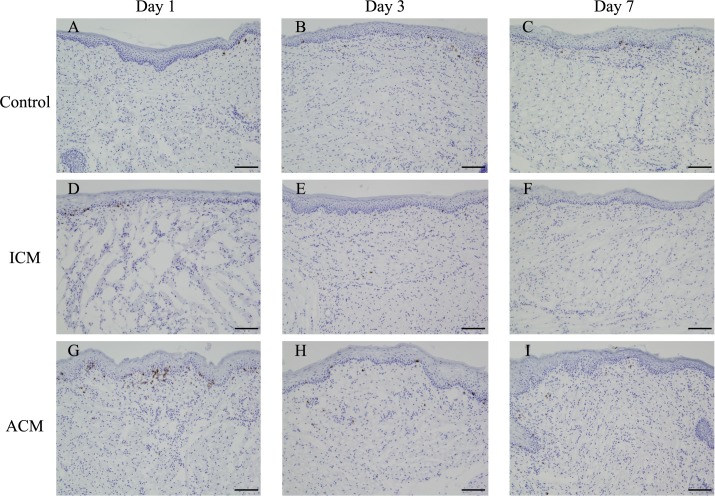
Immunohistochemical analyses of accumulating T cells in the oral mucosa of Ni-induced allergic mice. Frozen buccal mucosa sections were stained with anti-CD3 antibodies at days 1, 3, and 7 after challenge. At day 1 after challenge, infiltrating CD3-positive T cells were present in the epithelial basal layer and the upper dermis in ACM mice (G). Scale bar = 10 μm.

**Fig 6:**
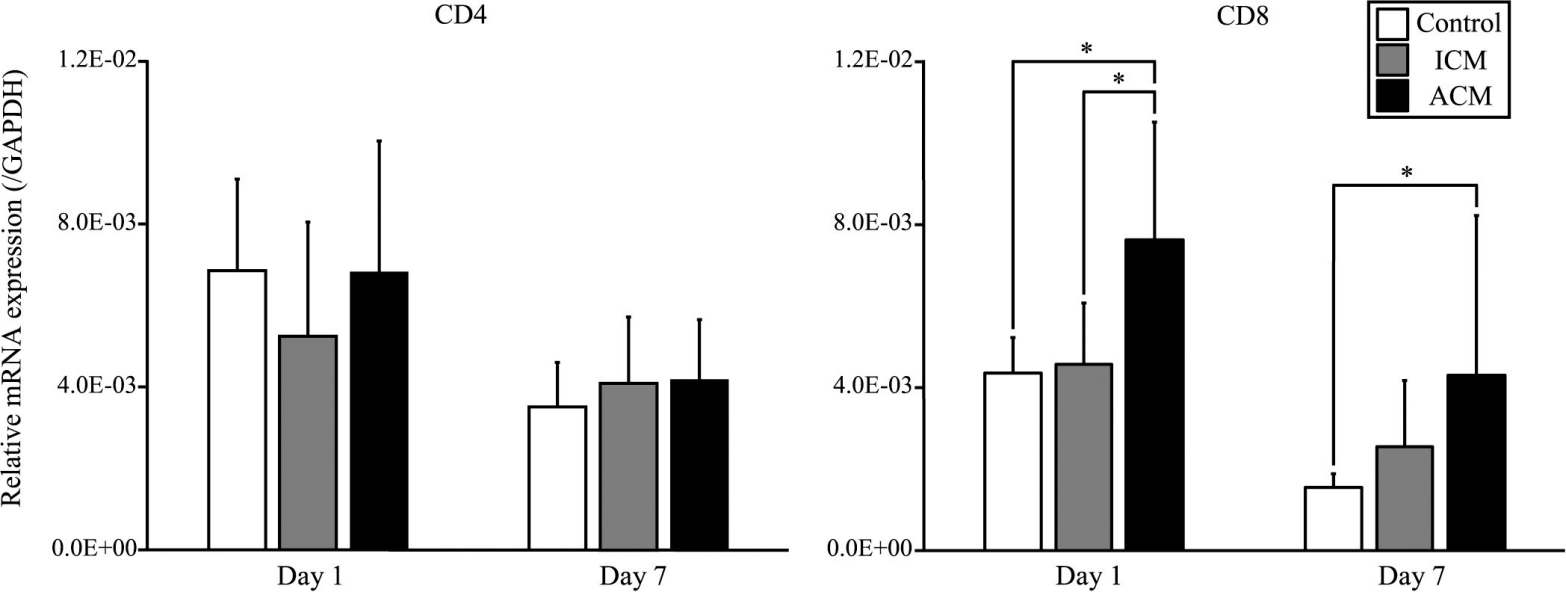
mRNA expression of T cell phenotypes in the oral mucosa of Ni-induced allergic mice. mRNA expression levels of CD4 and CD8 in the buccal mucosa were assessed at days 1 and 7 after challenge. GAPDH gene expression was used as an internal control. Buccal mucosa mRNA levels of CD8 were significantly higher in ACM mice compared with control and ICM mice at day 1 after challenge. However, CD4 levels were not significantly different between control, ICM, and ACM mice. Bars and error bars indicate the mean plus standard deviation. Statistical significance was tested by Dunn's test (* *p* < 0.05).

To verify whether T cells infiltrated into the inflamed oral mucosa in ACM mice, we performed IHC analysis of CD3 and qPCR analysis of CD4 and CD8 expressions. IHC staining showed that CD3-positive T cells markedly infiltrated into the epithelial basal layer and the upper dermis of ACM mice at 1 day after challenge compared with control and ICM mice (Fig 5G). CD8 levels were significantly higher in ACM mice than in control and ICM mice at 1 day after challenge (Fig 6). However, CD4 levels were not significantly different between the oral mucosa of ACM, ICM, and control mice (Fig 6).

### Expression levels of T cell-related cytokines, cytotoxic granules, and regulatory T cell transcription factors in the oral mucosa of Ni-induced ICM and ACM *BALB/cAJcl* mice

**Fig 7:**
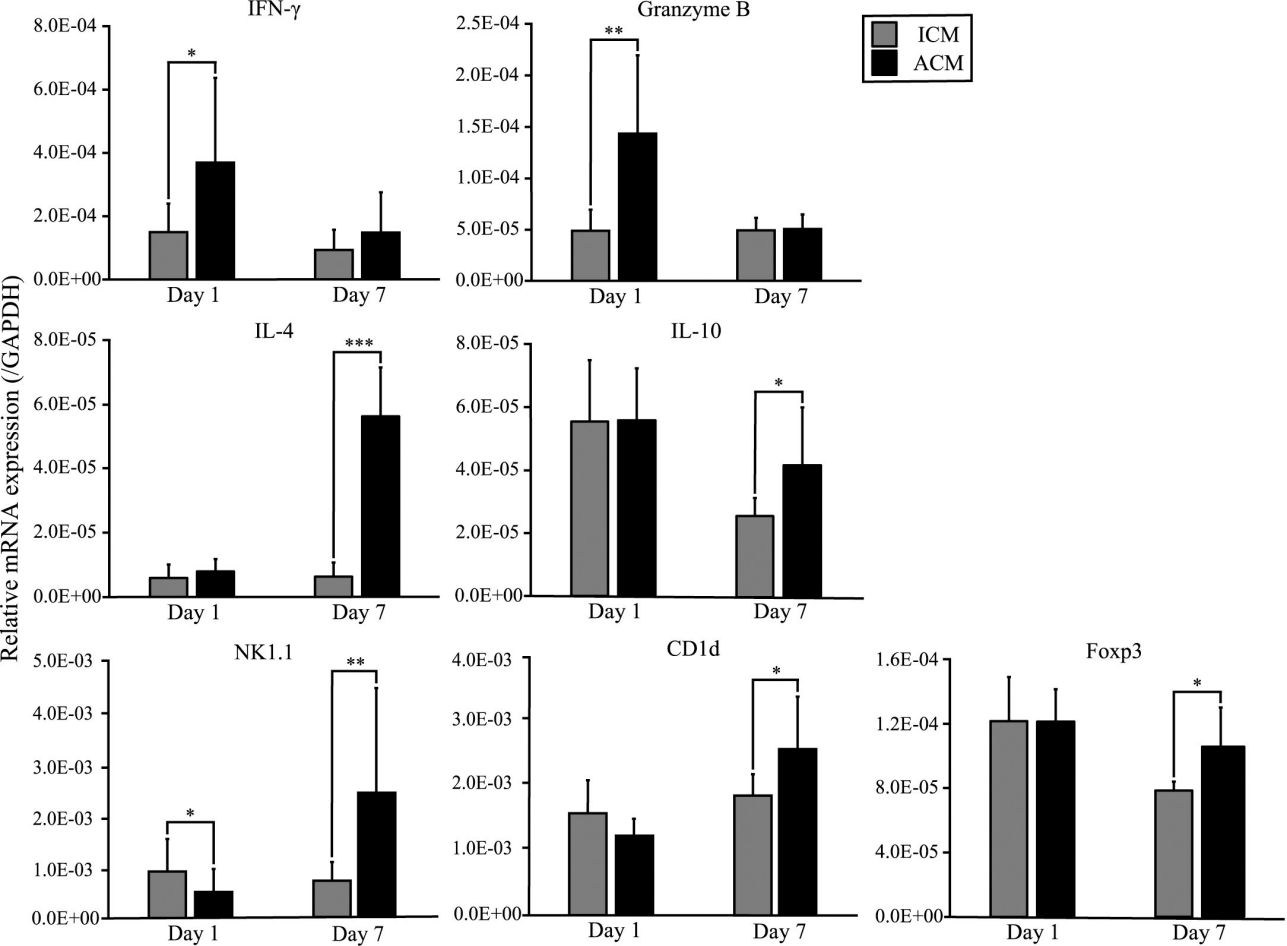
mRNA expression of T cell-related cytokines, cytotoxic granules and Foxp3 in ICM and ACM mice. The mRNA expression levels of IFN-γ, IL-4, IL-10, Granzyme B, NK1.1, CD1d, and Foxp3 in the buccal mucosa were assessed at days 1 and 7 after challenge. GAPDH gene expression was used as an internal control. At day 1 after challenge, the buccal mucosa mRNA levels of IFN-γ and Granzyme B were significantly higher in ACM mice compared with ICM mice. IL-4, NK1.1, CD1d, IL-10, and Foxp3 levels were significantly higher in ACM mice compared with ICM mice at day 7 after challenge. Bars and error bars indicate the mean plus standard deviation. Statistical significance was tested by the Mann–Whitney *U-*test (* *p* < 0.05, ** *p* < 0.01, *** *p* < 0.001).

We compared the expression levels of a Th1-related gene (IFN-γ), Th2-related genes (IL-4 and IL-10), cytotoxic granules (Granzyme B), NKT cell markers (NK1.1 and CD1d), and transcription factors of regulatory T cells (Foxp3) by qPCR analysis of the oral mucosa of Ni-induced ICM and ACM mice. In ACM mice, the expression levels of IFN-γ and Granzyme B were significantly higher than in ICM mice at 1 day after challenge (Fig 7). Moreover, the expression levels of IL-4, NK1.1, CD1d, IL-10, and Foxp3 were significantly higher in ACM mice than in ICM mice at 7 days after challenge (Fig 7).

### TCR repertoire usage in the oral mucosa and cervical lymph nodes of Ni-induced ICM and ACM *BALB/cAJcl* mice at day 1 after challenge

**Fig 8:**
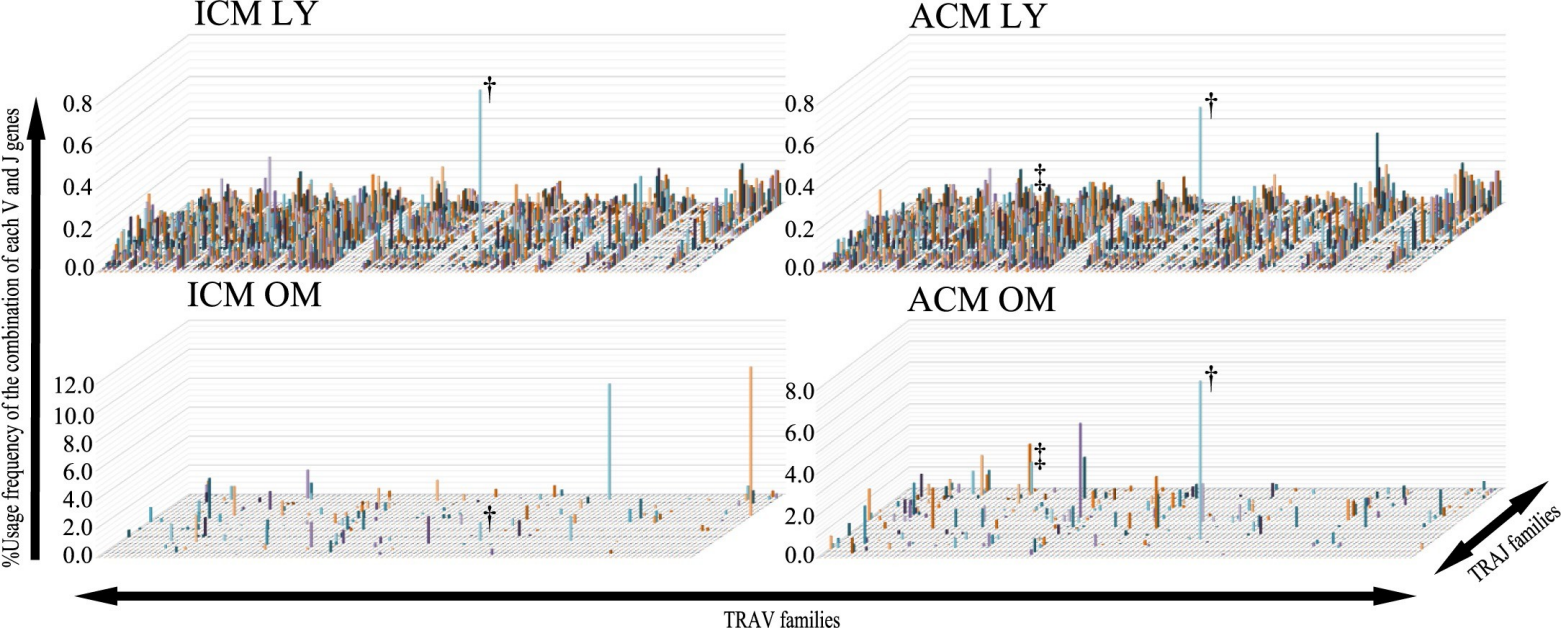
3D Graphic analysis of the TRAV repertoire in ICM and ACM mice. TRAVJ repertoires were analyzed from the buccal mucosa and cervical lymph nodes of ICM and ACM mice at day 1 after challenge by NGS. 3D images of the TCR repertoire bearing a combination of TRV with TRJ genes show the skewing of T cells infiltrating the oral mucosa and cervical lymph nodes of ICM and ACM mice at day 1 after challenge. In the cervical lymph nodes, the percentage usage of *Trav11d-Traj18* was high in ICM and ACM mice. The percentage usage of *Trav11d-Traj18* was higher in the buccal mucosa of ACM mice compared with the buccal mucosa of ICM mice. †; TRAs bearing *Trav11d-Traj18*, and ‡; TRAs bearing *Trav6-6-Traj57*. OM; oral mucosa, and LY; cervical lymph nodes.

To determine the TCR repertoire of T cells that contribute to Ni allergy in the oral mucosa, we analyzed the TRV and TRJ expression levels in the inflamed oral mucosa and cervical lymph nodes of ICM and ACM mice at 1 day after challenge by NGS-based TCR repertoire analysis. In the cervical lymph nodes, the percentage usage of *Trav11d-Traj18* was high in ICM and ACM mice (Fig 8). Of note, the percentage usage of *Trav11d-Traj18* was higher in the oral mucosa of ACM mice compared with the oral mucosa of ICM mice (Fig 8). The *Trav11d* gene is a subgroup of the *Trav11* gene and mouse invariant natural killer T (iNKT) cells express a TRA encoded by *Trav11-Traj18* gene segments [27].

Next, we investigated the common CDR3 sequences of TRA between ICM and ACM mice. Sequence analyses showed that shared TRA sequences contained a high proportion of the invariant TRAs indicative of iNKT cells in the cervical lymph nodes of ICM and ACM mice (S1 Fig). Interestingly, the proportion of the invariant TRAs indicative of iNKT cells in the oral mucosa of ACM mice was higher than in the oral mucosa of ICM mice and normal spleens (Table 1). Furthermore, T cells bearing *Trav6-6-Traj57* with common CDR3 sequences were detected in the oral mucosa and cervical lymph nodes of ACM mice; however, this clone was rarely detected in ICM mice and normal spleens (Table 1). In the TRB sequences, there was no shared TRB clone in either the inflamed oral mucosa or the cervical lymph nodes of ICM and ACM mice (S2 Fig).

**Table 1:** Distributions of read % of iNKT cells and T cells bearing *Trav6-6-Traj57* in ICM, ACM mice and normal spleen.

Common CDR3 sequences of TRA	CDR3	Mean Reads%				
		ICM OM	ACM OM	ICM LY	ACM LY	Spleen
iNKT cells bearing *Trav11d-Traj18*	CVVGDRGSALGRLHF	1.14±0.92%	6.60±4.35%	0.67±0.24%	0.57±0.08%	4.13±1.04%
T cells bearing *Trav6-6-Traj57*	CALGDQGGSAKLIF	0.00%	2.21±1.21%	0.01±0.01%	0.03±0.01%	0.00±0.01%

Amino acid sequences of CDR3 regions of TRA were obtained from the oral mucosa and cervical lymph nodes of ACM and ICM mice. Spleens were obtained from normal *BALB/cAJcl* mice. The proportion of iNKT cells and T cells bearing *Trav6-6-Traj57* were calculated from the total number of detected TRA clones. The proportion of iNKT cells in the oral mucosa of ACM mice was higher than in the oral mucosa of ICM mice and normal spleens. T cells bearing *Trav6-6-Traj57* were detected in the oral mucosa and cervical lymph nodes of ACM mice, but rarely detected in ICM mice or normal spleens. OM; oral mucosa, LY; cervical lymph nodes.

### Comparison of swelling between the oral mucosa and footpad of Ni-induced allergic *BALB/cAJcl* mice

**Fig 9:**
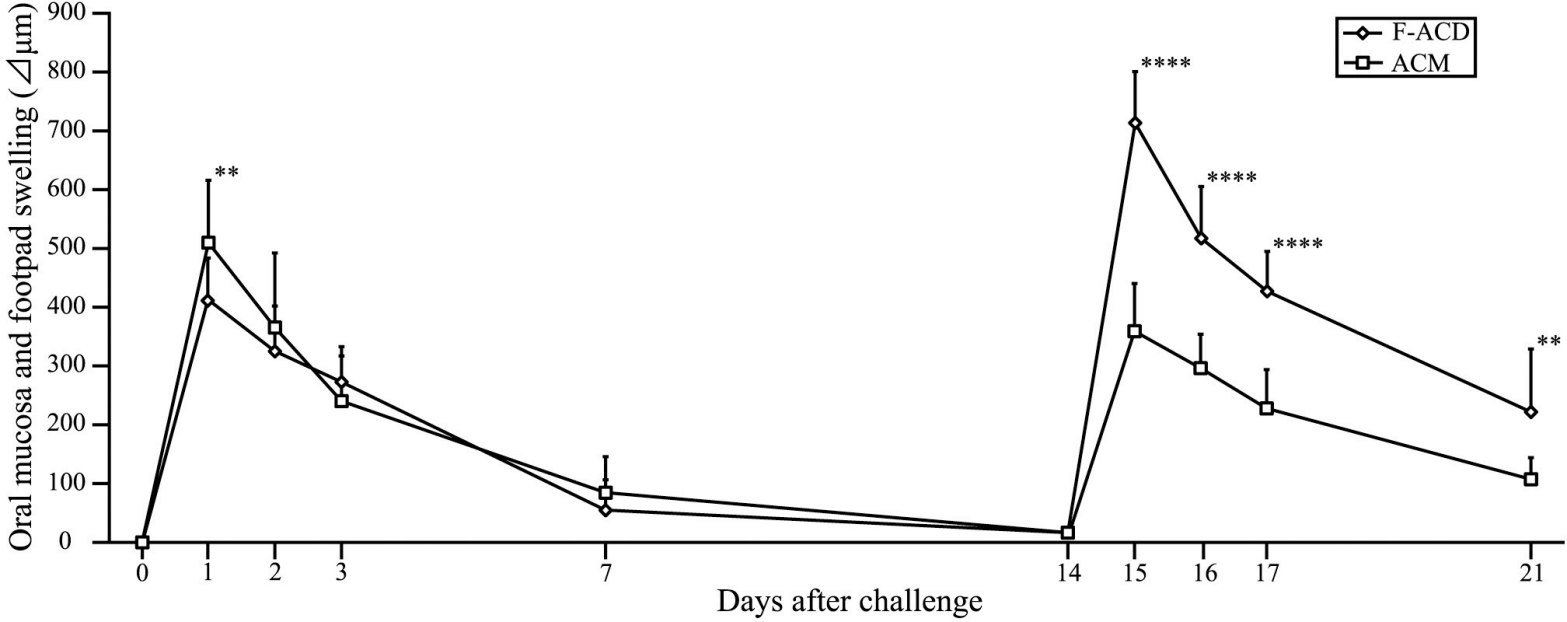
Comparison of swelling between the oral mucosa and footpad of Ni-induced allergic mice. The swelling of ACM and F-ACD mice at 1, 2, 3, and 7 days after the first and second challenges. At day 0 and day 14 after sensitization, the challenge was performed in ACM and F-ACD mice. Swelling at day 1 after the first challenge was significantly increased in ACM mice compared with F-ACD mice. Swelling from 1 to 7 days after the second challenge was significantly increased in F-ACD mice compared with ACM mice. Bars and error bars indicate the mean plus standard deviation. Statistical significance was tested by the Mann–Whitney *U-*test (** *p* < 0.01, **** *p* < 0.0001).

To compare responses in the elicitation phase after oral mucosa or footpad challenge, we compared the swelling of ACM and F-ACD mice after the first and second challenges. Swelling at 1 day after the first challenge was significantly increased in ACM mice compared with F-ACD mice (Fig 9). However, swelling from 1 to 7 days after the second challenge was significantly increased in F-ACD mice compared with ACM mice (Fig 9). In the second challenged F-ACD mice, the footpad was markedly swollen compared with the first challenged F-ACD mice (Fig 9). In contrast to the F-ACD mice, the buccal area swelling of ACM mice was reduced at the second challenge (Fig 9).

### Immune response of Ni-induced intraoral metal contact allergy in NKT cell deficient mice

**Fig 10:**
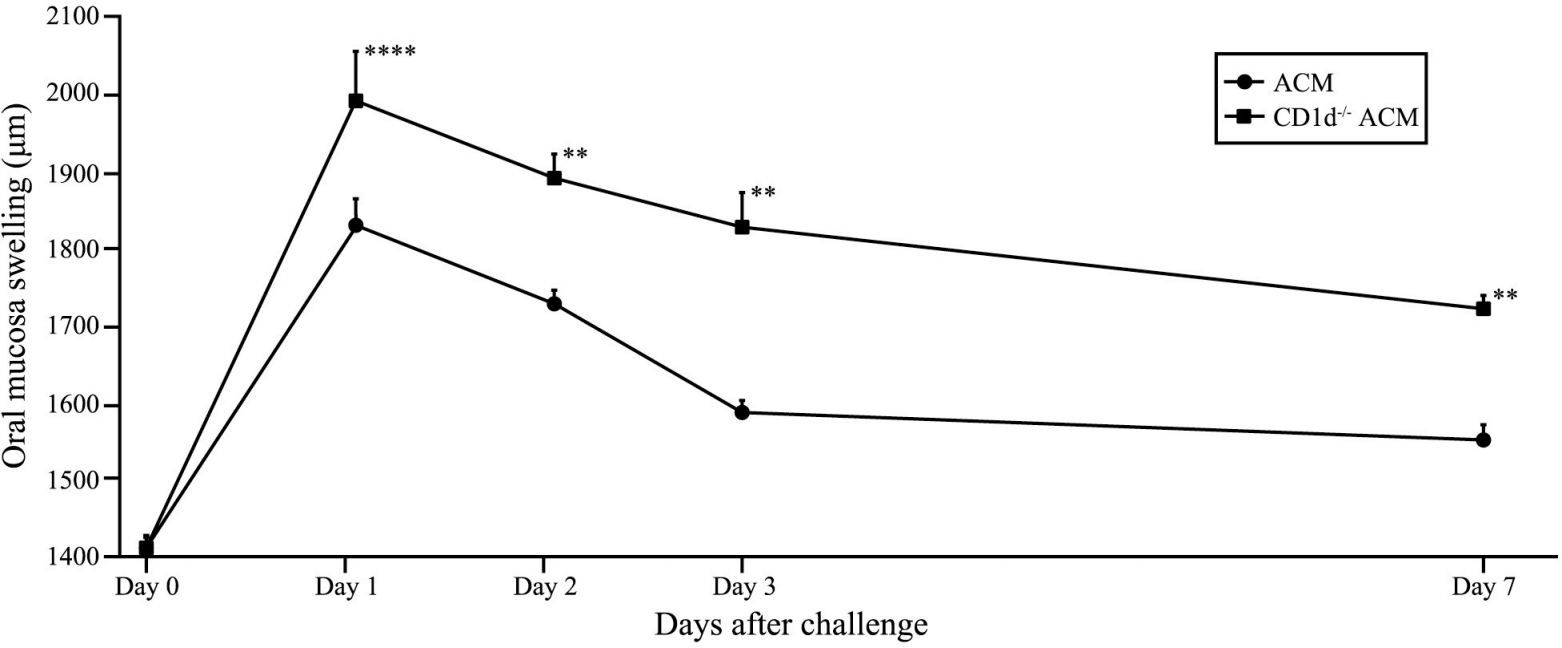
Swelling of the oral mucosa in *CD1d*^*-/-*^ Ni-induced allergic mice. Buccal area swelling from days 1 to 7 after challenge was significantly higher in *CD1d*^*-/-*^ ACM mice compared with ACM mice. Bars and error bars indicate mean plus standard deviation. Statistical significance was tested by the Mann–Whitney *U-*test (** *p* < 0.01, **** *p* < 0.0001).

**Fig 11:**
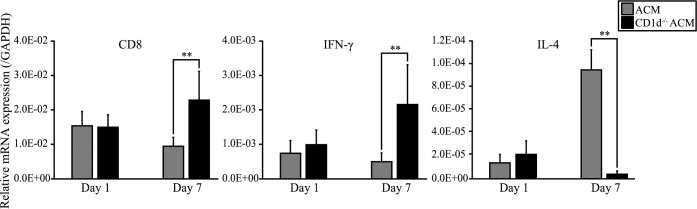
mRNA expressions of CD8, IFN-γ and IL-4 in *CD1d*^*-/-*^ ACM mice. The mRNA expression levels of CD8, IFN-γ and IL-4 in the buccal mucosa were assessed at days 1 and 7 after challenge. GAPDH gene expression was used as an internal control. At day 7 after challenge, the buccal mucosa mRNA levels of CD8 and IFN-γ were significantly higher in *CD1d*^*-/-*^ ACM mice compared with ACM mice. IL-4 was significantly higher in ACM mice compared with *CD1d*^*-/-*^ ACM mice at day 7 after challenge. Bars and error bars indicate the mean plus standard deviation. Statistical significance was tested by the Mann–Whitney *U-*test (** *p* < 0.01).

To verify the functional role of NKT cells in Ni-induced intraoral metal contact allergy, we compared swelling of the oral mucosa and the expression levels of CD8, IFN-γ and IL-4 in the oral mucosa of *C57BL/6* ACM mice and *CD1d*^*-/-*^ ACM mice. From 1 to 7 days after challenge, swelling of the buccal mucosa was significantly higher in *CD1d*^*-/-*^ ACM mice compared with ACM mice (Fig 10). In *CD1d*^*-/-*^ ACM mice, the expression levels of CD8 and IFN-γ were significantly higher than in ACM mice at 7 days after challenge (Fig 11). Moreover, the expression level of IL-4 was significantly higher in ACM mice than in *CD1d*^*-/-*^ ACM mice at 7 days after challenge (Fig 11).

## Discussion

This study reports the successful establishment of a murine model of Ni-induced intraoral metal contact allergy, and indicates that DTH reactions in the oral mucosa were evoked by Ni. In addition, we identified antigen-specific T cells expressing common TCR repertoires in the oral mucosa and cervical lymph nodes of Ni-induced ACM mice. Previous studies reported that chemicals induced DTH responses in the oral mucosa of ACM mice [17–19]. However, an animal model for intraoral metal contact allergy has not been established; therefore, metal-specific immune responses in the oral mucosa have not been elucidated. This is the first report regarding the establishment of a murine model of intraoral metal contact allergy and the characterization of metal-specific immune responses in the oral mucosa.

In this study, we generated Ni-induced ICM and ACM in the oral mucosa of mice. ICM is a non-specific inflammatory response to various external stimuli that arises because of activated innate immunity to the direct exposure to chemicals or metals without prior sensitization [28]. ACM corresponds to a DTH response with an acquired immunity mechanism that induces allergen-specific T cells [28]. In this murine model, swelling of the oral mucosa from 6 h to 3 days after challenge was significantly increased in ACM mice compared with control and ICM mice. In ACM mice, swelling of the oral mucosa reached a maximum at 1 day after challenge. Previous studies reported that the most pronounced inflammatory reactions of DTH in murine oral mucosa were observed at 1 day after elicitation [17–19]. We previously reported that footpad swelling reached a maximum at 1 day after challenge in ACD mice [15]. Our results showing swelling caused by the DTH reaction were consistent with these previous studies.

Histopathological analysis showed dense inflammatory cell infiltration, swelling of the oral mucosal epithelium, and apparent epidermal spongiosis in ACM mice at 1 day after challenge. At 3 days after the challenge, the numbers of inflammatory cells in the epithelial basal layer and upper dermis were reduced, and at 7 days after challenge, swelling of the oral mucosal epithelium and epidermal spongiosis were diminished in ACM mice. Spongiosis is a characteristic histopathological feature observed in acute eczema [29]. Inflammatory reactions in the control and ICM mice were relatively diminished compared with ACM mice after challenge. IHC staining showed that F4/80-positive macrophages and CD3-positive T cells markedly infiltrated into the epithelial basal layer and upper dermis of ACM mice at 1 day after challenge compared with control and ICM mice. Thereafter, at 3 and 7 days after challenge, F4/80-positive macrophages and CD3-positive T cells were present at very low numbers in ACM mice. Previous studies reported that DTH reactions in the oral mucosa have the hallmarks of skin DTH reactions with T cells and macrophages [17, 19]. Our results were consistent with these previous studies and suggest that allergic inflammation in the oral mucosa was initiated by the response of macrophages to Ni, followed by T cell infiltration into the inflamed oral mucosa after antigen presentation by macrophages. Moreover, dendritic cells in the oral mucosa have a higher T cell stimulating capacity compared with skin dendritic cells *in vitro* [30, 31]. We previously reported that the infiltration of T cells into the footpad skin of Ni-induced ACD mice reached a maximum at 7 days after challenge [13]; however, the infiltration of T cells reached a maximum at 1 day after challenge in the oral mucosa of Ni-induced ACM mice. This discrepancy may be explained by the capacity of dendritic cells to activate T cells.

Metal allergy can be elicited by the infiltration of either CD4 or CD8 T cells into inflammatory sites depending on the pathway by which antigen is processed [32–34]. In this murine model, the mRNA expression of CD8 was significantly higher in ACM mice compared with control and ICM mice at 1 day after challenge. A previous study reported many CD8-positive T cells were recruited into the inflamed oral mucosa of a murine model of DTH [18]. Therefore, CD8-positive T cells may have a proinflammatory role in the elicitation phase of DTH in the oral mucosa. Furthermore, we compared the expression levels of IFN-γ, IL-4, IL-10, Granzyme B, and Foxp3 in the oral mucosa of Ni-induced ICM and ACM mice. The expression levels of IFN-γ and Granzyme B were significantly higher in ACM mice compared with ICM mice at 1 day after challenge. IFN-γ produced by Th1 cells activates macrophages and cooperates with CD8-positive T cells to amplify the inflammatory response [35]. Moreover, it was suggested that CD8-positive T cells secrete IFN-γ as an effector molecule to induce metal allergy and DTH responses to chemicals [34, 36]. A previous study reported that skin obtained from Ni allergy patients showed an enhanced expression of Granzyme B [29]. CD8-positive T cells are cytotoxic T cells that kill target cells by releasing cytotoxic granules. Our results indicate that IFN-γ is the main effector cytokine of Ni allergy in the oral mucosa. Furthermore, the apoptosis of keratinocytes induced by macrophages and CD8-positive T cells might be implicated in the pathogenesis of Ni allergy in the oral mucosa. The ectodermal oral mucosa reacts similarly to skin and upon antigen exposure inflammation occurs. The oral mucosa has a degree of immune privilege and T-cell tolerance is induced similar to that in the intestinal tract [37–39]. In this study, the expression levels of IL-4, IL-10, and Foxp3 were significantly higher in ACM mice than in ICM mice at 7 days after challenge. A previous study reported significantly low numbers of IL-4 and IL-10 producing cells in the peripheral blood of Ni allergy patients [40]. Another study suggested that the lack of a Th2 inhibitory effect increased the detrimental effects of Th1 cells in ACD [41]. Consistent with these previous studies, our results suggested that IFN-γ expression was markedly increased when ACM mice developed severe allergic inflammation, whereas IL-4 and IL-10 expressions were markedly increased when allergic inflammation was diminished in ACM mice. This suggests that the Ni allergic immune response in the oral mucosa may shift from Th1 to Th2 bias between days 1 and 7 after challenge. A previous study suggested that IL-10-producing CD4-positive regulatory T cells isolated from the skin and peripheral blood of nickel-allergic patients were primarily involved in the regulation of ACD by inhibiting the maturation and functions of dendritic cells [42]. Results from ACM mice at 7 days after challenge indicated that excessive Ni allergy in the oral mucosa might be suppressed by regulatory T cells.

In the present study, we used NGS to analyze TRAV and TRAJ expression levels and the common CDR3 sequences of TRA. This NGS method enabled a comprehensive quantitative analysis of TCR repertoires at a clonal level and the identification of antigen-specific T cells in DTH responses. In this study, we investigated the common T cell clone in the inflamed oral mucosa and cervical lymph nodes of ICM and ACM mice. As a result, we detected a high proportion of iNKT cells and T cells bearing *Trav6-6-Traj57* in the oral mucosa and cervical lymph nodes of Ni-induced ACM mice. However, there were no other shared T cell clones in either the inflamed oral mucosa or the cervical lymph nodes of ACM mice. iNKT cells are characterized by the expression of an invariant TRA encoded by *Trav11*(*Vα14*)*-Traj18*(*Jα18*) in mice and *TRAV10*(*Vα24*)*-TRAJ18* (*Jα18*) in humans [43, 44]. NKT cells were identified in the lesional skin of contact dermatitis (including Ni allergy) patients [45]. Recent studies suggested that NKT cells have a critical inductive role during the sensitization and elicitation phase of ACD mice [46–48]. In our Ni-induced ACM mice, we detected a high frequency of iNKT cells in the oral mucosa and cervical lymph nodes (S1 Fig). We recently showed that iNKT cells participate in the control of immune responses of metal allergy [49]. Our results suggest that iNKT cells control the immune responses of Ni allergy in the oral mucosa by bridging between the innate immune response in the sensitization phase and adaptive immunity in the elicitation phase. Interestingly, T cells bearing *Trav6-6-Traj57* were detected in the oral mucosa and cervical lymph nodes in ACM mice, but this clone was rarely detected in ICM mice and normal spleens. The involvement of T cells bearing *Trav6-6-Traj57* in the pathogenesis of DTH has not been reported. Our results suggest that T cells bearing *Trav6-6-Traj57* may participate in the development of Ni-induced intraoral metal contact allergy.

To compare the responses in the elicitation phase after oral mucosa and footpad challenge, we compared the swelling in ACM and F-ACD mice. Swelling at 1 day after the first challenge was significantly increased in ACM mice compared with F-ACD mice. We previously reported that CD4-positive T cells and CD4 CD8 double-negative T cells accumulated in the footpad skin of Ni-induced ACD mice at 7 days after the third challenge [13]. However, in the present study, CD8-positive T cells accumulated in the oral mucosa of Ni-induced ACM mice at 1 day after the first challenge. Ni allergy in the oral mucosa induced a more rapid and severe inflammation than Ni allergy in the footpad skin. This result may reflect differences in the dilatation and density of capillaries, permeability of materials, and accumulated T cells. Moreover, in F-ACD mice after the second challenge, the footpad was markedly swollen compared with F-ACD mice after the first challenge. However, in contrast to F-ACD mice, swelling of the buccal area of ACM mice was reduced at the second challenge. These results indicate that excessive Ni allergy in the oral mucosa is also suppressed by regulatory T cells at the second challenge.

We previously reported metal-specific immune responses in murine models of Ni, Pd, Cr, and Ti-induced allergic contact dermatitis [13–16]. These studies suggested that accumulated metal-specific T cells at the site of allergic inflammation differ based on the type of metal. We observed Ni-specific T cells bearing *Vα14Jα18/Vβ8*.*2*, Pd-specific T cells bearing *Vα18-1/Vβ8–2*, Cr-specific T cells bearing *Vα11-1/Vβ14–1* and Ti-specific T cells bearing *Trav1-Traj33* [13–16]. Interestingly, we identified iNKT cells in the lymphocytic infiltrates at a high frequency during the elicitation phase in Ni, Cr, and Ti allergies [13, 15, 16]. However, the functional role of iNKT cells in intraoral metal contact allergy has not been reported. In the present study, the expression levels of NK1.1 and CD1d were significantly higher in ACM mice compared with ICM mice at 7 days after challenge. These results indicate that iNKT cells increase between days 1 and 7 after challenge in ACM mice. Moreover, swelling of the buccal mucosa from days 1 to 7 after challenge was significantly higher in *CD1d*^*-/-*^ ACM mice compared with ACM mice, and the expression level of IL-4 was decreased and the expression levels of CD8 and IFN-γ were increased in *CD1d*^*-/-*^ ACM mice compared with ACM mice at 7 days after challenge. These results suggest that IL-4 is secreted by iNKT cells in ACM mice at 7 days after challenge, and that IFN-γ is secreted by CD8-positive effector T cells in ACM mice at 1 day after challenge. A previous study suggested that ACD was suppressed by iNKT cells, the interaction between dendritic cells and CD8 T cells initiated the activation of iNKT cells, and that IL-4 production by iNKT cells required the presence of hapten-specific CD8-positive effector T cells that released IFN-γ [50]. Our results were consistent with this previous study. Therefore, excessive Ni allergy in the oral mucosa might be suppressed by IL-4 produced from iNKT cells and IL-10 produced from regulatory T cells. NKT cells in mice are mainly CD4 single-positive or CD4 CD8 double-negative. In this study, we found that the mRNA expression of CD8 was significantly higher in ACM mice compared with control and ICM mice at 1 day after challenge. Moreover, results of additional experiments using iNKT cell-deficient mice indicated that Ni allergy in the oral mucosa might be suppressed by iNKT cells. Therefore, T cells bearing *Trav6-6-Traj57* detected in the oral mucosa and cervical lymph nodes of ACM mice at 1 day after challenge might be the metal-specific T cells in Ni-induced oral allergic mice, which are thought to be CD8-positive effector T cells. In conclusion, we established a novel murine model of Ni-induced allergy in the oral mucosa, and showed that iNKT cells and T cells bearing *Trav6-6-Traj57* might be involved in the immune responses of Ni-induced intraoral metal contact allergy. Our novel mouse model is useful for understanding the pathological roles of T cells in intraoral metal contact allergy. Further studies using this mouse model will contribute to the diagnosis of intraoral metal contact allergy as well as the development of new treatments to control metal-specific T cells in the oral mucosa.

## Supporting information

S1 Fig Top 40 ranking read numbers of TRA clonotypes in ICD and ACD mice. Ranking of TRA clonotype frequency indicated a high proportion of iNKT cells in the cervical lymph nodes of ICM and ACM mice. In the buccal mucosa, the proportion of iNKT cells in ACM mice was higher than in ICM mice. A high proportion of T cells bearing *Trav6-6-Traj57* were detected in the buccal mucosa and cervical lymph nodes of ACM mice.
(TIF)

S2 Fig Top 40 ranking read numbers of TRB clonotypes in ICD and ACD mice. There was no shared TRB clone between the oral mucosa and cervical lymph nodes in ICM and ACM mice.
(TIF)
