# Supplementary figures and images for: Characterization of T cell receptors in a novel murine model of nickel-induced intraoral metal contact allergy
Source: PLoS One. 2018 Dec 17;13(12):e0209248. doi: 10.1371/journal.pone.0209248 (PMC6296741; doi:10.1371/journal.pone.0209248)

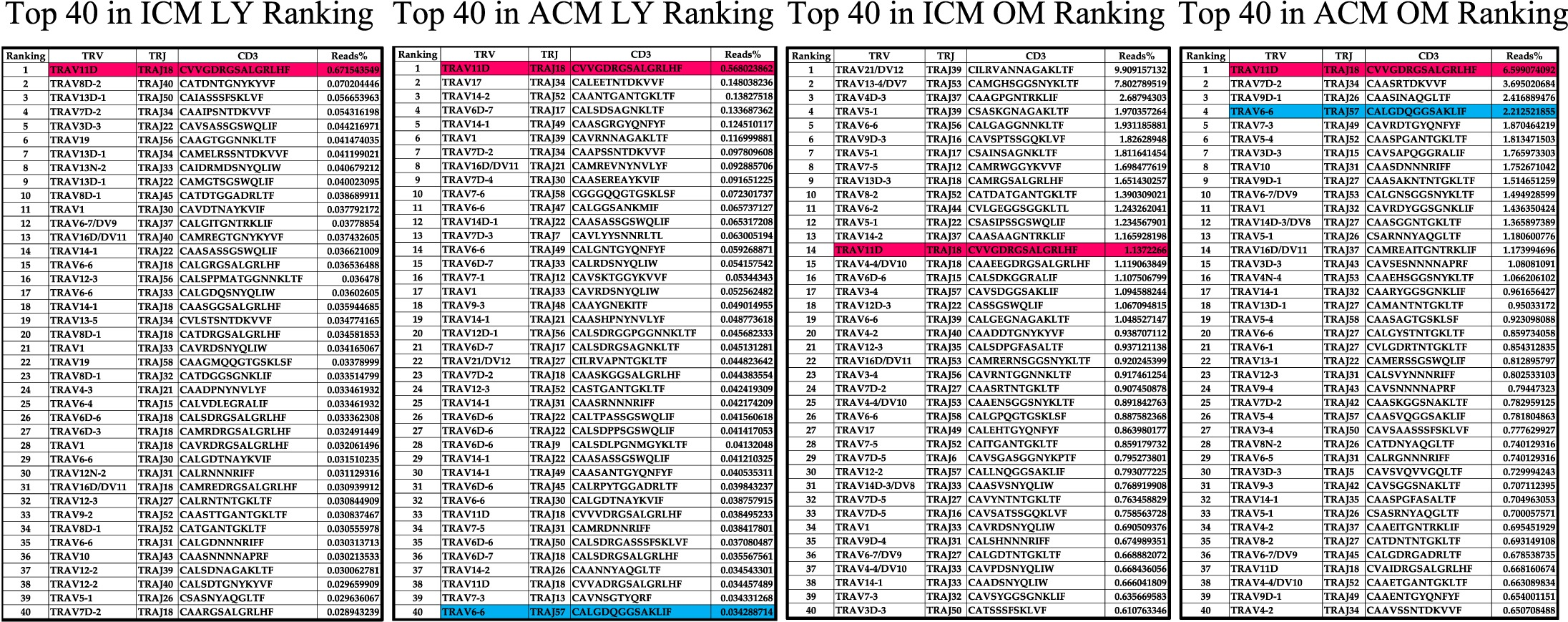

Supplement: S1 Fig — Ranking of TRA clonotype frequency indicated a high proportion of iNKT cells in the cervical lymph nodes of ICM and ACM mice. In the buccal mucosa, the proportion of iNKT cells in ACM mice was higher than in ICM mice. A high proportion of T cells bearing Trav6-6-Traj57 were detected in the buccal mucosa and cervical lymph nodes of ACM mice. (TIF) [file pone.0209248.s001.tif]

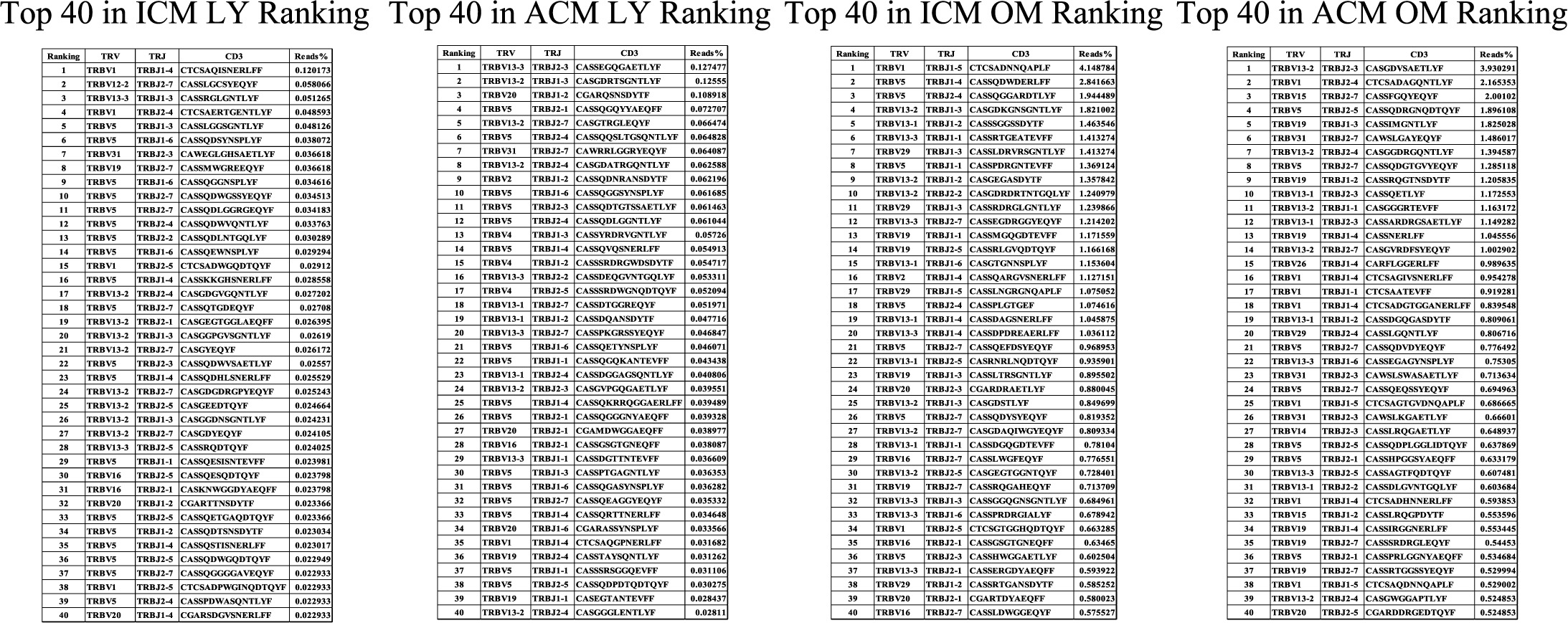

Supplement: S2 Fig — There was no shared TRB clone between the oral mucosa and cervical lymph nodes in ICM and ACM mice. (TIF) [file pone.0209248.s002.tif]
